# Socioeconomic Inequalities in COVID-19 Incidence During Different Epidemic Phases in South Korea

**DOI:** 10.3389/fmed.2022.840685

**Published:** 2022-03-08

**Authors:** Dae-sung Yoo, Minji Hwang, Byung Chul Chun, Su Jin Kim, Mia Son, Nam-Kyu Seo, Myung Ki

**Affiliations:** ^1^Department of Public Health, Korea University Graduate School, Seoul, South Korea; ^2^Veterinary Epidemiology Division, Animal and Plant Quarantine Agency, Gimcheon, South Korea; ^3^BK21FOUR R&E Center for Learning Health Systems, Korea University, Seoul, South Korea; ^4^Department of Preventive Medicine, College of Medicine, Korea University, Seoul, South Korea; ^5^Department of Emergency Medicine, College of Medicine, Korea University, Seoul, South Korea; ^6^Department of Preventive Medicine, School of Medicine, Kangwon National University, Chuncheon, South Korea; ^7^Department of Non-Benefits Management, National Health Insurance Service/Health Insurance Policy Research Institute, Wonju, South Korea

**Keywords:** COVID-19, inequality, mobility, SARS-CoV2, social distancing, socioeconomic, spatial analyses

## Abstract

**Objective:**

Area-level socioeconomic status (SES) is associated with coronavirus disease 2019 (COVID-19) incidence. However, the underlying mechanism of the association is context-specific, and the choice of measure is still important. We aimed to evaluate the socioeconomic gradient regarding COVID-19 incidence in Korea based on several area-level SES measures.

**Methods:**

COVID-19 incidence and area-level SES measures across 229 Korean municipalities were derived from various administrative regional data collected between 2015 and 2020. The Bayesian negative binomial model with a spatial autocorrelation term was used to estimate the incidence rate ratio (IRR) and relative index of inequality (RII) of each SES factor, with adjustment for covariates. The magnitude of association was compared between two epidemic phases: a low phase (<100 daily cases, from May 6 to August 14, 2020) and a rebound phase (>100 daily cases, from August 15 to December 31, 2020).

**Results:**

Area-level socioeconomic inequalities in COVID-19 incidence between the most disadvantaged region and the least disadvantaged region were observed for nonemployment rates [RII = 1.40, 95% credible interval (Crl) = 1.01–1.95] and basic livelihood security recipients (RII = 2.66, 95% Crl = 1.12–5.97), but were not observed for other measures in the low phase. However, the magnitude of the inequalities of these SES variables diminished in the rebound phase. A higher area-level mobility showed a higher risk of COVID-19 incidence in both the low (IRR = 1.67, 95% Crl = 1.26–2.17) and rebound phases (IRR = 1.28, 95% Crl = 1.14–1.44). When SES and mobility measures were simultaneously adjusted, the association of SES with COVID-19 incidence remained significant but only in the low phase, indicating they were mutually independent in the low phase.

**Conclusion:**

The level of basic livelihood benefit recipients and nonemployment rate showed social stratification of COVID-19 incidence in Korea. Explanation of area-level inequalities in COVID-19 incidence may not be derived only from mobility differences in Korea but, instead, from the country's own context.

## Introduction

Since the first case, reported in December 2019 in China, the severe acute respiratory syndrome coronavirus-2 (SARS-CoV2; COVID-19) pandemic has caused unprecedented global challenges due to rapid interpersonal transmission. This virus causes symptoms ranging from mild, such as sore throat and fever, to severe pneumonia resulting in death (1). Due to a higher transmission rate than other coronaviruses (reproduction ratio: 2.44–4.18) and a high proportion of asymptomatic infectious people (2), the global pandemic has grown significantly, causing nearly 271.4 million cases with 5.3 million deaths (as of 16th December 2021) according to the World Health Organization (3).

In Korea, since the first case of COVID-19 in a person who visited China was identified on January 20, 2020, multiple clustered outbreaks associated with religious followings, call centers, and courier services led to a surge in the number of disease occurrences; this was followed by enhanced strict counteractive measures, including social distancing, that were enforced by health authorities, which reduced the weekly average number of cases to single digits (4). However, due to increased outdoor activities, large-scale gatherings during the holiday season and seasonality, the number of newly infected cases grew dramatically to more than 1,000 cases daily, mostly driven by a substantial increase in infections in the capital region, where 25.92 million people live within 11,851.26 km^2^, one of the most densely populated areas in the world.

Historically, disadvantaged people have been highly vulnerable to emerging infectious diseases, especially when they become a persistent epidemic (5). In recent studies on COVID-19, historic evidence showed that socioeconomically vulnerable individuals were more likely to have higher incidence and case-fatality rates of COVID-19 (6, 7). This indicates that underlying socioeconomic gradients are strongly associated with the distribution of incidence and fatality rates of COVID-19, due to variations in personal hygiene, access to testing and treatment, compliance level with social distancing policy, and the ability to work remotely (8). In recent studies regarding COVID-19 in the United States, low-income individuals were less able to reduce their mobility or maintain social distancing, indicating that economic activity is highly associated with behavioral responses to social distancing policy (9, 10).

In addition to individual socioeconomic vulnerability, area-level socioeconomic disadvantages have consistently been associated with COVID-19 incidence. Area-level socioeconomic status (SES) tends to depend on territory-based communities that characterize human society because of a shared socioeconomic basis, commonality in available services, living culture, and lifestyle (11). Area-level socioeconomic measures have been identified in various ways and typically measured using an aggregate variable (e.g., median household income) or a composite measure (e.g., deprivation index). Each measure represents a unique contribution to the socioeconomic association. Specifically, associations with COVID-19 were consistently observed for median household income (12, 13) and minor ethnicity (1, 14, 15) but findings for deprivation index (16, 17) and unemployment rate (13, 14) were inconsistent, indicating that area-level SES measures have different values across time and place and that how they are measured is important (18).

Individuals from lower SES areas are more likely to be infected for various reasons; however, in most studies, the primary cause was the lack of mobility reduction resulting in the inability to maintain social distancing. However, the mediating role of mobility was advocated in other studies to explain area-level socioeconomic inequalities in COVID-19 infection based on the high correlation between area-level SES and mobility reduction (10, 16, 19, 20). Despite wide acceptance of the explanation, studies in which the underlying relationship was investigated using both measures are scarce. Thus, firm empirical evidence is lacking on whether the effect of area-level SES on COVID-19 incidence depends on the level of mobility. This concept may be particularly relevant in countries like Korea, where socioeconomic inequalities in COVID-19 incidence may not be straightforward because affluent areas are also a central business place.

As noted below, Korea had been undergone a relatively lower level of COVID-19 incidence compared to other countries (21). Nevertheless, a better understanding of regional disparity in COVID-19 incidence is a huge challenge because it is essential to monitor the pattern of spread into subsegment of the population, let alone the incidence from the entire population. Thus, we investigated the socioeconomic inequalities in COVID-19 incidence at the level of a primary administrative unit of local government in Korea, using a diverse range of socioeconomic indicators including a mobility measure. In this study, we investigated (1) whether area-level socioeconomic measures are associated with COVID-19 incidence at the municipality level; (2) whether the associations' differences in the association between socioeconomic inequalities and COVID-19 incidence in two different epidemic phases with disparate social distancing enforcement; and (4) whether socioeconomic inequalities in COVID-19 infection are mainly due to mobility differences.

## Materials and Methods

### Study Base

**Figure 1:**
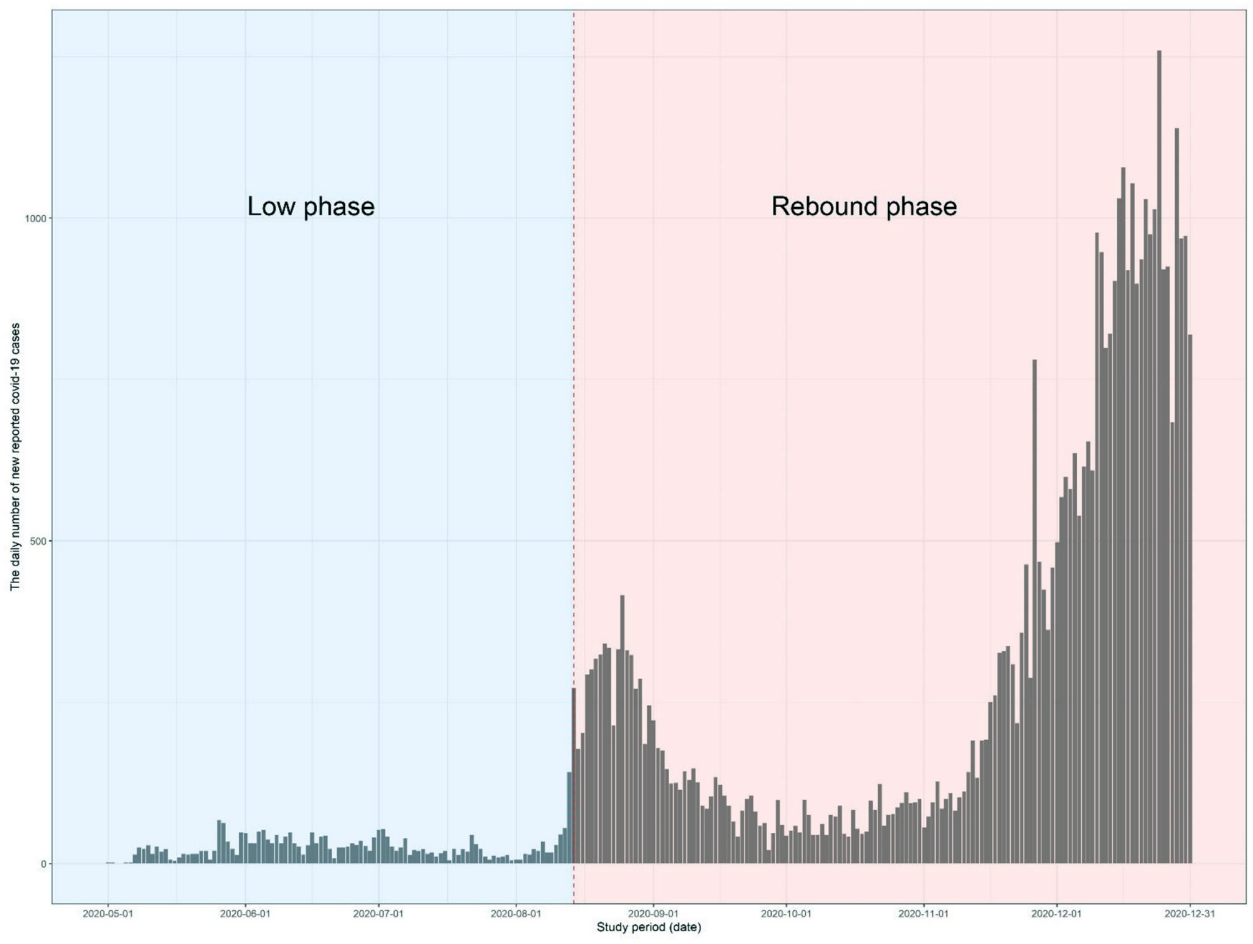
The epidemic curve of coronavirus disease 2019 (COVID-19) in the Korea during the study period (from May 1 to August 14, 2020, for low phase and from August 15 to December 31, 2020, for rebound phase). The gray bar represents the daily number of newly reported COVID-19 cases.

Overall, Korea experienced favorable outcomes of COVID-19 compared with other countries in terms of incidence and mortality through the pandemic and the study period (21, 22). To evaluate the effects of socioeconomic inequalities on COVID-19 incidence at different epidemic levels, the epidemic period was divided into two phases based on the daily number of cases and the accompanying social distancing intensity level as shown in Figure 1: low phase (from May 6 to August 14, 2020) in which less than 100 mean daily cases were confirmed with the eased social distancing regulation (level 1) and rebound phase (from August 15 to December 31, 2020) in which more than 100 mean daily cases were reported with stricter distancing imposed (level 2). Because the early phase of the epidemic was induced by a specific religious congregation concentrated in very limited municipalities, the starting time point in this study was March 5, 2020, to ensure the validity of the results (23).

The social distancing level enforced by the Korean government was classified into two levels during the study period through the guidelines underwent several changes afterward. For example, under level 2 social distancing, the use of face masks in public became mandatory, social gatherings with more than a certain number of individuals were prohibited and restaurants must be closed after a specific time point but without movement restriction. Lower social distancing regulation (i.e., level 1) began from May 6 to August 14, 2020, and stricter social distancing measure (i.e., level 2) was enacted from August 15, 2020.

### Socioeconomic Status Measures and Covariates

The information on COVID-19 incidence as an outcome variable was collected from 229 municipalities and compiled from the KCDC and the local administration's official websites. As listed in Table 1, six area-level (i.e., municipality) socioeconomic factors were used to investigate the effects of inequality on the incidence of COVID-19 in Korea. The indicators were classified into two subcategories, SES, and economic activity, based on the corresponding attributes. SES measures included the following: national insurance contributions as the proxy of area-specific income level; material deprivation index (MDI); nonemployment rate; the proportion of basic livelihood security recipients; financial autonomy of the area. Economic activity included mobility at risk. Data on national insurance contributions in the first quarter of 2020 were obtained from the Korean National Health Insurance Services. MDI for each area was a composite index derived from the sum of standardized Z-scores for eight measures based on data from the national population and housing census conducted by the National Statistical Office of Korea; the proportion of nonemployed males, manual laborers, households under the minimum housing standard, nonsecured housing tenure, nonapartment housing, lower educational level (≤middle school), single-parent household and school dropouts between 9 and 24 years of age (24). The higher the MDI score, the more the area is deprived. The nonemployment rate was calculated as the proportion of individuals who were unemployed or out of the labor force (e.g., early retirement, studying, and disability) between 30 and 64 years of age (25), based on data from the National Population and Housing Census in 2015. The proportion of basic livelihood security recipients at the area level in 2019 was retrieved from the Korea Social Security Information Service. Financial autonomy for each area was defined as a ratio of total revenue generation to the total expenditure per municipality as provided by the Korean Statistical Information Service for 2019. To determine the socioeconomic strata of socioeconomic factors, those continuous values of socioeconomic factors were converted into quintiles of their distribution (i.e., each stratum accounted for 20% of the number of municipalities) (26).

**Table 1:** Description of the variables used in the study with the source of data.

**Category**	**Variable (units)**	**Description**	**Source (period)**
Outcome	COVID-19 (No. of Cases)	The sum number of cases of COVID-19 by municipality	Korean center for disease control and local administration (May 6, 2020 – December 31, 2020)
Socioeconomic status	National insurance contributions (US Dollar)	Average amount of personal national insurance contributions per month by municipality	Korean national health insurance services (1st quarter of 2020)
	Material deprivation index (Z-score)	Composite index derived from the sum of standardized Z-scores for eight measures; the proportions of nonemployed males, manual class, households under the minimum housing standard, insecure housing tenure, living apartment, nonapartment housing, lower educational achievement (≤middle school), single-parent household, school drop-out between 9 and 24. Data were driven from the National population and housing census by the National Statistical Office of Korea by municipality	National population and housing census of the National Statistical Office of Korea (2015)
	Nonemployment rate (%)	The proportion of individuals who were unemployed or out of the labor force aged from 30 to 64 years	National population and housing census of the National Statistical Office of Korea (2015)
	Basic livelihood security recipient (%)	The total number of households receiving basic livelihood security over total number of households according to national basic living security act	Korea social security information service (2019)
	Financial autonomy (%)	The ratio of revenue generation to total expense by municipality	Korean statistical information service (2020)
Economic activity	Mobility at risk (Z-score)	The volume of public transportation times works related movement divided by total amount of traffic volume	Korean Transport Institute (2018)
Covariates	Population density (No. of inhabitants /km^2^)	Human population on resident registry over the land size estimated	Korean statistical information service (2020)
	Median age (years)	Median age of residents in registry by municipality	Korean statistical information service (2020)
	Health care workforce (No. of health care workers per 1,000 persons)	The sum of total number of medical doctors, dentists, pharmacist, and health care worker	Korean statistical information service (2020)

In addition, the municipality-specific economic activity variable, including the volume of traffic for mobility at risk represented by a Z-score, was added. Mobility at risk was equal to the proportion of the traffic volume of work-related movement utilizing public transportation, which was calculated by multiplying the volume of public transportation and the volume of works-related traffics (e.g., commuting to work and field trips). This variable was obtained from a transportation survey conducted by the Korean Transport Institute in 2018. Finally, three covariates, namely, municipality-specific median age, population density, and the number of healthcare workers per 1,000 inhabitants, were used to adjust for the demographic composition and the local health care capacity of the areas in our analysis. The variables were derived from the data obtained from the Korean Statistical Information Service for 2020. The data in our study were extracted from open sources, which are aggregated by administrative subdivisions. Therefore, do not contain any information that is indicative of information about personal or household level. The Institutional Review Board (IRB) of Korea University granted an exemption for this study (IRB exemption number: KUIRB-2020-0297-01).

### Statistical Analyses

Several steps of the analytical process were applied to examine socioeconomic inequalities in COVID-19 incidence. Due to the nature of spatial data, spatial autocorrelation on the SES variables and three covariates were examined using Global Moran's I test before investigating the association between SES measures and COVID-19 incidence. The statistical significance of the Global Moran's I was estimated with 999 simulations. Following identification of the presence of spatial autocorrelation in socioeconomic indicators, the association between socioeconomic measures and COVID-19 incidence was estimated as an incidence rate ratio (IRR) using a spatial negative binomial model with marten correlation function for spatial correlation term (Model 1). To account for potential confounding factors, adjustment was initially made for three covariates (i.e., median age, population density, and healthcare workforce at the area-level) (Model 2). In addition, we conducted a regression with a further adjustment for economic activity to evaluate the mediating effect of mobility on the association between area-level SES and COVID-19 incidence (Model 3).

We built a Bayesian generalized linear model to estimate the posterior marginal distribution of IRR of each SES measure. Because the observed incidence rate by the municipality, used as the outcome of interest, was overdispersed, it was modeled as a negative binomial random variable with overdispersed variance instead of Poisson regression. In addition, the Besag, York, and Mollié (BYM) model was used to account for spatial autocorrelation of residuals by adding a spatial random effect using intrinsic conditional autoregressive (iCAR) function and extra residual term for spatially independent variation that was independent, identical, and normally distributed as follows:


$$\begin{array}{l} Y_{i}\sim NB\left(\pi_{i},r_{i}\right),Y_{i}:\;Number\;of\;COVID-19\;cases\\ by\;municipality\;i\\ \pi_{i}=\frac{r_{i}}{r_{i}+\lambda_{i}},E\left(Y_{i}\right)=\lambda_{i}\\ log\;\left(\lambda_{i}\right)={\alpha +\log\left({\text{populatio}n}_{i}\right)+\beta }_{1}\times {SES}_{i,k}\\ +\overset{N}{\underset{j=2}{\sum }}\beta_{j}\times covariate_{i}+u_{i}+\varepsilon_{i}\\ u_{1:229}\sim ICAR\;(W,\sigma_{u}^{2})\\ \varepsilon \sim N\left(0,\sigma_{\varepsilon }^{2}\right)\\ u\;\sim N0,\;I-C^{-1}\;\times M,C=\gamma \times W,M=I\times \;\sigma_{u}^{2} \end{array}$$

where *u*_*i*_ is the conditional autocorrelation regression term, the covariance matrix of the parameters calculated based on the neighboring regions, ε_*i*_ is the nonspatial structured term, *u* is the spatial correlated random effect calculated by averaging neighboring random effects, *I* is the identity matrix, and *W* is the spatial weights matrix constructed by an inverse distance function with the exponents followed by row-standardized such that each row sums to 1 for interpretation of the parameters (27). The neighboring region at each municipality was defined as the administrative division located within the geographical distance that was not spatially correlated in a variogram generated using a Bayesian generalized linear model without the spatial correlation term. The spatial correlation parameter denoted as γ was set to 1.

The models were run with three chains with different starting values in which sampling values in the MCMC process with a burn-in of 4,000 iterations and a thinning rate of 10, and 50,000 iterations were used for each posterior distribution of parameters for SES and covariates. Convergence of the chains was assessed by visual inspection of the posterior distributions and computation of the Gelman–Rubin statistic. The Deviance Information Criterion (DIC) was used to measure and compare the goodness of fit for the model. The prior distribution for each parameter and hyperparameter is described in the Supplementary Material. R2WinBUGS R software package version 2.1 (28) with WinBUGS software version 1.4.3 was used to carry out given statistical approaches (29). The map presented in this study was created by Esri ArcGIS software version 10.8.1 using the South Korea map which is publicly available (30). All analyses were separately performed for two different phases of the COVID-19 pandemic; the low and rebound phases.

We repeated a similar analysis to estimate the relative index of inequality (RII) as a supplementary measure of inequalities in the COVID-19 incidence rate at the area level. RII is a commonly used measure of health inequalities that summarizes the distribution of a health outcome measure against an SES as a relative difference of the least and most deprived subgroups (31). RII in this study corresponds to the relative risk of the incidence for COVID-19 in the lowest and the highest socioeconomic strata and, therefore, is directed by changes in two strata (Supplementary Material). The RII was also estimated using a spatial negative binomial model with marten correlation function for spatial correlation term, 95% CI was estimated by bootstrap. RII estimation was made as follows.


$$\begin{array}{l} Y_{i}\sim NB\left(\pi_{i},r_{i}\right),Y_{i}:\;Number\;of\;COVID-19\;cases\\ by\;municipality\;i\\ \pi_{i}=\frac{r_{i}}{r_{i}+\lambda_{i}},E\left(Y_{i}\right)=\lambda_{i}\\ \log\left(\lambda_{i}\right)={\alpha +\log\left(population_{i}\right)+\beta }_{1}\times {SES}_{i,k}\\ +\overset{N}{\underset{j=2}{\sum }}\beta_{j}\times covariate_{i}+u_{i}+\varepsilon_{i}\\ u_{1:229}\sim ICAR\;(W,\sigma_{u}^{2})\\ \varepsilon \sim N\left(0,\sigma_{\varepsilon }^{2}\right)\\ u\;\sim N\left(0,\;{(I-C)}^{-1}\;\times M\right),C=\gamma \times W,M=I\times \sigma_{u}^{2} \end{array}$$

where *x*_*i*_ denotes the mid-point of municipality *i* in socioeconomic class *k* with number 1 assigned to the highest class of SES, as opposed to the lowest strata. The mid-point was derived for each SES class. In addition, SES variables are likely to be mutually correlated. Thus, Spearman's correlation coefficient between two paired SES variables was estimated to exclude the correlated combinations for subsequent multivariate analyses.

## Results

### Overview of COVID-19 Incidence and Socioeconomic Characteristics

The COVID-19 epidemic in Korea showed two distinctive phases in terms of the incidence level over the study period as illustrated in Figure 1. In the low phase (from May 6 to August 14, 2020), 2,906 cases were reported in 141 municipalities with 28.8 daily new cases for 100 days, in which no escalating pattern was observed in the epidemic curve. In contrast, in the rebound phase (from August 15 to December 31, 2020), 40,545 cases were reported in 224 municipalities with 291.7 daily cases for 139 days, in which two distinctive peaks were observed in the epidemic curve.

**Figure 2:**
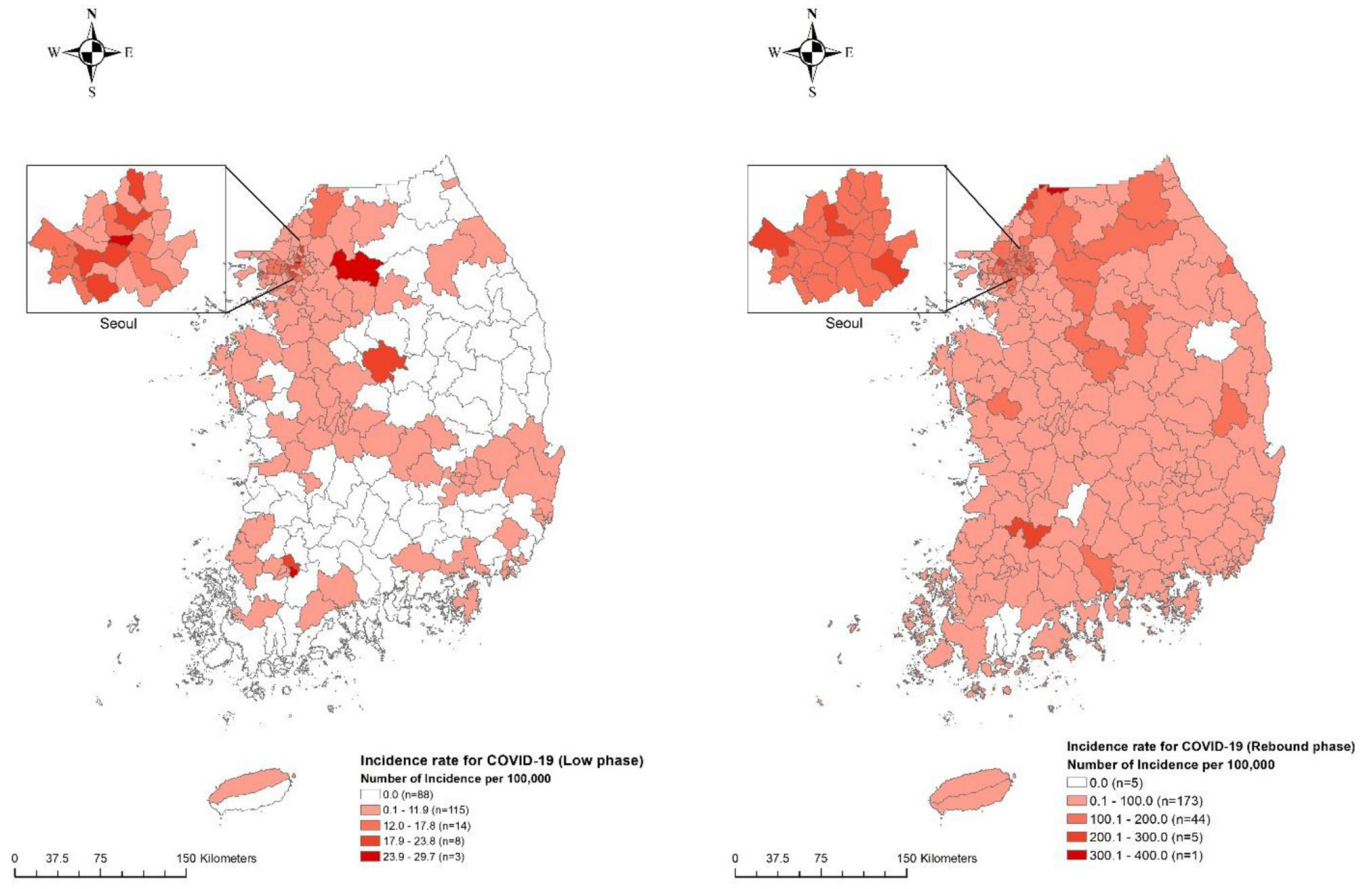
Geographical distribution of municipality-specific incidence rate for COVID-19 in Korea between two epidemic phases. The number of incidences for COVID-19 per 100,000 inhabitants at the municipality level is denoted by five different color levels in the low phase of COVID-19 (**left**) and rebound phase of COVID-19 (**right**). Darker red shedding represents the highest strata, whereas brighter red shedding denotes the lowest strata along with white color representing noncase.

Geographically, a significant difference was observed in the area-level COVID-19 incidence rate as shown in Figure 2. On average, 12.7 cases were reported per area [minimum (min) – maximum (max) = min – max = 0–127 cases] in the low phase and 177.1 cases (min – max = 0–1,653 cases) were reported in the rebound phase. The majority of COVID-19 cases were reported in the Seoul metropolitan area (81.8% in the low phase and 72.8% in the rebound phase) where 50.28% of the total Korean population resides within 11,851.26 km^2^ (11.8% of the land size of Korea). The average nonemployment rate was 13.9 and 4.9% of households received basic livelihood security (Table 2). All variables, in particular, economic activity, showed significant spatial autocorrelation in the Global Moran's I test indicating that the association of those variables with COVID-19 should be measured with consideration of spatial autocorrelation.

**Table 2:** Overview of socioeconomic status measures, economic activity variables, and covariates for 229 municipalities in Korea.

**Variables**				**Descriptive statistics**		
	**Mean**	**SD**	**Min**	**Max**	**CV**	**Global morans‘I^†^**
Socioeconomic status (unit)						
National insurance contributions (US dollars)	43.18	10.58	27.76	100.43	0.25	0.71 (0.001)
Material deprivation index (Z-score)	0.00	5.61	−12.41	14.59	-	0.48 (0.001)
Nonemployment rate (%)	13.86	3.12	4.53	24.10	0.23	0.34 (0.001)
Basic livelihood security recipient (%)	4.48	1.57	1.27	9.79	0.26	0.62 (0.001)
Financial autonomy (%)	24.96	12.60	6.60	68.00	0.33	0.57 (0.001)
Economic activity						
Mobility at risk (Z-score)	0.00	1.00	−1.48	2.84	-	0.87 (0.001)
Covariates						
Population Density (No. of inhabitant/km^2^)	45.78	87.66	0.20	516.19	1.92	0.36 (0.001)
Median Age (years)	47.47	6.08	37.20	61.00	0.13	0.49 (0.001)
Health care workforce (No. of workers per 1,000 persons)	8.21	6.87	2.57	54.02	0.84	0.23 (0.002)

*SD, standard deviation; Min, minimum; Max, maximum; CV, coefficient of variance = standard deviation/mean*.

† *The significance of the statistics of Global Morans‘I was estimated with 999 simulations, expressed in parenthesis*.

**Figure 3:**
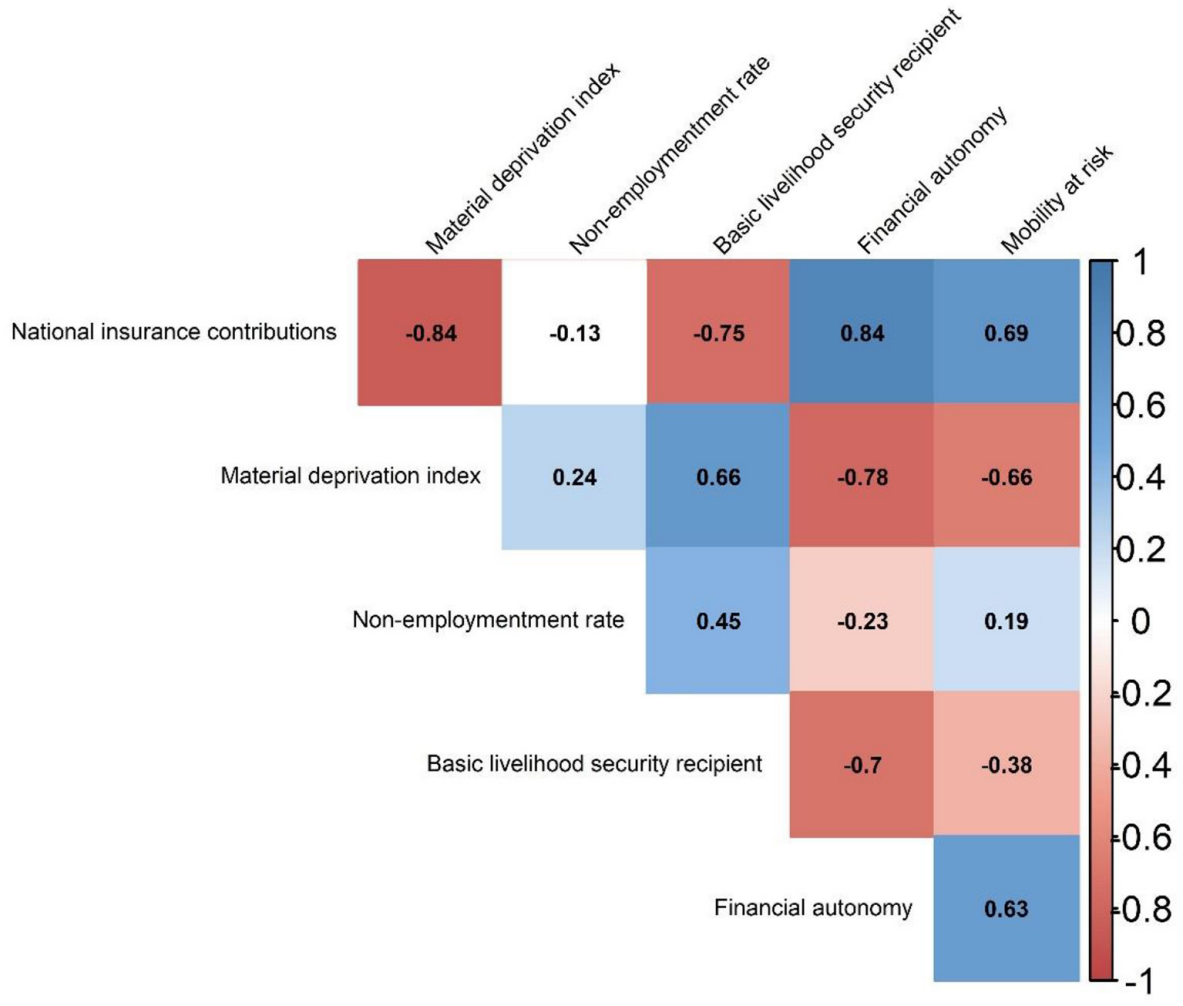
Correlation plot between socioeconomic status and economic activity variables. The number inside the cell corresponded to Spearman correlation coefficient estimates. The intensity of correlation was expressed by colored gradient where dark blue represented one (a complete positive correlation) and dark red represented minus one (a complete negative correlation). All correlation coefficients estimates were statistically significant (*p* < 0.05).

Generally, socioeconomic measures were significantly correlated with each other (Figure 3) but heterogeneous in direction. For example, national insurance contributions as the proxy of personal income level had a negative correlation with indicators of social exclusion and poverty [e.g., MDI (Spearman coefficient = −0.84], the proportion of basic livelihood security recipients [Spearman coefficient = −0.75), and the nonemployment rate (Spearman coefficient = −0.13)]. Notably, a negative correlation of economic activity (i.e., mobility at risk) was observed with indicators of social exclusion and poverty such as the proportion of basic livelihood security recipients (Spearman coefficient = −0.38) but not with nonemployment rate (Spearman coefficient = 0.69), and a positive correlation of mobility at risk was shown with national insurance contribution (Spearman coefficient = 0.69), indicating economic activity (i.e., mobility at risk) was characteristic of affluent areas.

### Associations Between Area-Level Socioeconomic Measures and COVID-19 Incidence

**Figure 4:**
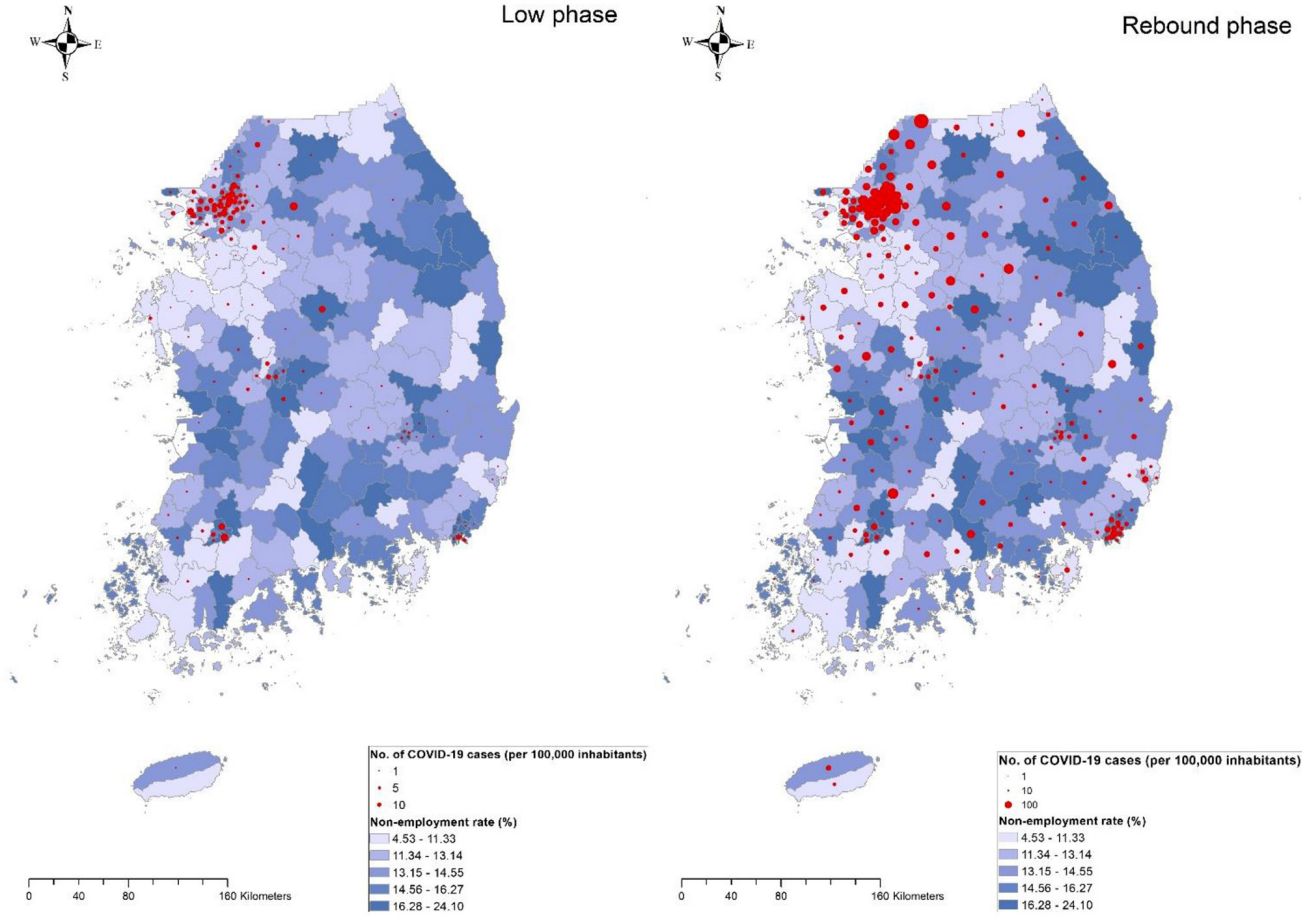
Geographical distribution of nonemployment rate coupled with COVID-19 incidence rate by 229 municipalities during the low phase of the epidemic (**left**) or the rebound phase (**right**). The size of the circle is proportional to the cumulative number of reported COVID-19 cases per 100,000 inhabitants during the corresponding period. Blue gradient represents the magnitude of the nonemployment rate.

**Figure 5:**
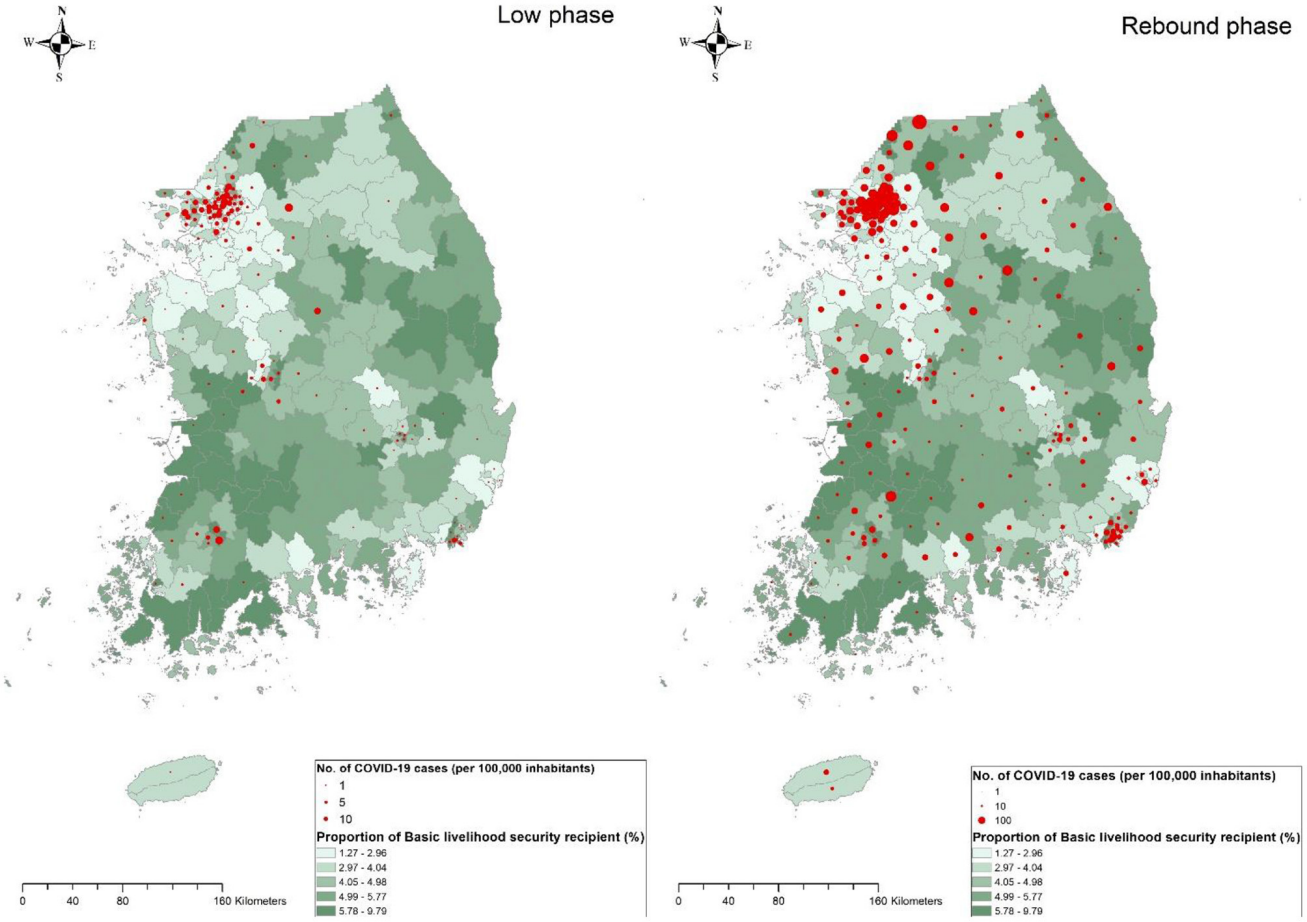
Geographical distribution of the proportion of basic livelihood security recipients with COVID-19 incidence rate by 229 municipalities during the low phase of the epidemic (**left**) or the rebound phase (**right**) The size of the circle is proportional to the cumulative number of reported COVID-19 cases per 100,000 inhabitants during the corresponding period. Green gradient represents the magnitude of the nonemployment rate.

Table 3 shows the estimation of IRR for the association between area-level SES measures and COVID-19 incidence using a Bayesian negative binomial regression. Overall, two area-level SES measures, nonemployment rate and the proportion of basic livelihood security recipients, were consistently associated with COVID-19 incidence based on unadjusted and adjusted modeling in the low and rebound phases. Specifically, in the low phase, the adjusted IRR corresponding to an increase in 1% of the nonemployment rate and the proportion of basic livelihood security recipients was estimated as 1.20 (95% credible interval (Crl) = 1.13–1.28) and 1.23 (95% Crl = 1.07–1.40), respectively (Model 2). In the rebound phase, the same SES measures presented inconsistence association with COVID-19 incidence. For example, the nonemployment rate showed a significantly negative association with COVID-19 incidence adjusted for only covariates (model 2), but for both covariates and mobility at risk (model 3), while the proportion of basic livelihood security recipients had an only univariate association with COVID-19 incidence (model 1) (Figures 4, 5).

**Table 3:** Incidence rate ratios for the association between socioeconomic status and economic activity and incidence for COVID-19 over the low and rebound phase in 229 municipalities in Korea.

**Variables**	**Low phase** **(no. of cases = 2,906)**			**Rebound phase** **(no. of cases = 40,545)**		
	**Model 1** ^†^	**Model 2** ^‡^	**Model 3^§^**	**Model 1** ^†^	**Model 2** ^‡^	**Model 3^§^**
Socioeconomic status						
National insurance contributions	1.01 (0.99, 1.03)	1.00 (0.98, 1.02)	-	1.01 (1.00, 1.02)	1.07 (0.82,1.40)	-
Material deprivation index (Z-score)	0.99 (0.95, 1.02)	0.98 (0.92, 1.04)	-	0.99 (0.97, 1.01)	1.00 (0.97,1.02)	-
Nonemployment rate	1.11 (1.06, 1.17)	1.20 (1.13, 1.28)	1.61 (1.09, 1.25)	1.02 (0.99, 1.05)	1.05 (1.02, 1.08)	1.02 (0.99, 1.06)
Basic livelihood security recipient	1.10 (1.02, 1.18)	1.23 (1.07, 1.40)	1.16 (1.02, 1.32)	1.04 (1.02, 1.06)	1.35 (0.93, 1.93)	1.04 (0.98, 1.09)
Financial autonomy	1.00 (0.98, 1.01)	0.98 (0.97, 1.00)	-	1.00 (1.00, 1.01)	1.00 (1.00, 1.01)	-
Economic activity						
Mobility at risk	1.69 (1.23, 2.35)	1.67 (1.26, 2.17)	1, 59 (1.22, 2.06)	1.23 (1.05, 1.46)	1.28 (1.14, 1.44)	1.26 (1.13, 1.41)
Covariates						
Population density	1.00 ^¶^ (1.00, 1.00 ^¶^)	-	-	1.00 ^¶^ (1.00, 1.00 ^¶^)	-	-
median age	0.99 (0.95, 1.03)	-	-	0.99 (0.97, 1.00)	-	-
Health care workforce	1.02 (1.01, 1.04)	-	-	1.01 (1.00, 1.02)	-	-

*The incidence rate ratio (IRR) was estimated using a Spatial and Bayesian negative binomial model with marten correlation function and BYM for spatial correlation term, 95% confidence interval was estimated by bootstrap, denoted in the parenthesis*.

† *Model 1: unadjusted model*.

‡ *Model 2: socioeconomic indicators were remained to estimate the associations, adjusting for covariates (human density, median age, and health care workforce)*.

§ *Model 3: two significant variables in Model 2 were retained to estimate the associations, adjusting for covariates from Model 2+ mobility at risk, separately. In turn, the incidence rate ratio for mobility at risk returned two estimates for each of two corresponding socioeconomic status variables. The incidence rate ratio of mobility at risk in this table was given as an adjustment factor for basic livelihood security recipients variable*.

¶ *denotes a given value is >1*.

An economic activity indicator (i.e., area-level mobility at risk) was positively associated with COVID-19 incidence rate in both the low (IRR = 1.67, 95% Crl = 1.26–2.17) and rebound phases (IRR = 1.28, 95% Crl = 1.14–1.44). When assessing the mediation of mobility at risk in the association between two SES measures and COVID-19 incidence, the magnitude of the associations was attenuated but remained significant in the low phase, but no associations were observed in the rebound phase (Model 3). For instance, the area with a higher nonemployment rate had a higher risk of COVID-19 incidence in the low phase (IRR = 1.61, 95% Crl = 1.09–1.25) but independent associations were not observed in the rebound phase (IRR = 1.02, 95% Crl = 0.99–1.06). When assessed with RII, nonemployment rate and the proportion of basic livelihood recipients showed a similar pattern of associations with COVID-19 incidence (Supplementary Table).

## Discussion

In this study, a significantly positive association was found between area-level nonemployment rate and the population share of basic livelihood security recipients and COVID-19 incidence. However, area-level socioeconomic effects were stronger in the low phase when the prevalence of COVID-19 was low, with less strict governmental measures (Figures 4, 5). In other words, the strength of the association of those SES measures decreased as the level of COVID-19 incidence rate across the country increased. Similarly, the inequalities in the COVID-19 incidence rate concerning nonemployment and basic livelihood security recipients were significantly high in the low phases. In addition, higher mobility at risk, indicating active economic activity at the area level, increases the risk of COVID-19 incidence in both phases. In this context, when both area-level SES measures and mobility were simultaneously adjusted, SES measures remained significant in the low phase, suggesting they were independent of each other in the low-risk period. However, in the rebound phase, adjustment for economic activity variables showed no association between SES measures and COVID-19 incidence. Overall, partial existence of COVID-19 inequalities in some measures may have occurred as a result of counteraction between risk raising and lowering area-level effects (e.g., poor communities are less mobile).

Among five measures of area-level SES, the areas with a higher level of basic livelihood security recipients and nonemployment rate showed a higher risk of COVID-19 incidence; however, an association was not observed for other area-level socioeconomic measures. A partial observation of area-level socioeconomic inequalities in COVID-19 incidence differs from most previous studies from the United States (32), the United Kingdom (20), and Spain (33, 34), and is similar to a previous Korean study (35) in which no or a partial association was observed. Inconsistency in inequalities in COVID-19 incidence across measures may possibly be interpreted using the socioeconomic context of Korea. Korean government measures were impartially imposed regarding case-identifying processes, awareness of the process, access to COVID-19 testing, and contact tracing, which may provide a relatively equal chance of being diagnosed. Close supervision by national mandatory conduct systems was uniformly applied regardless of area-level SES. This universal approach is not exclusive to Korea, but the outcome may be proequity in countries with high levels of public support for strong governmental measures. In addition, inequalities may be greater in places where COVID-19 diagnostic testing is often delayed, and choosing which patient to care for first is an issue when the number of patients is overwhelming (36). In contrast, Korea has maintained a lower COVID-19 incidence by enhancing rigorous contact tracing and extensive testing with no discrimination, which may have minimized some forms of socioeconomic disparity across areas. Inconsistent inequalities may be also explained by different conceptualizations of the five area-level SES measures. Basic livelihood security recipients are mostly older adults, and the age composition of this measure better reflects diagnosed COVID-19 cases; the majority (35.1% in the low phase and 31.6% in the rebound phase) were older adults (≥60 years of age), according to the Korea Central Disease Control Headquarters (http://ncov.mohw.go.kr/en/). This finding is in agreement with an individual-based Korean study in which higher SES was associated with higher COVID-19 incidence in the older population, and both higher and lower SES were associated with the younger population (37). Similarly, the area-level nonemployment rate largely depends on the proportion of individuals who are not in the labor force. However, the national health insurance premium and financial autonomy address directly the income level of the working population. The deprivation index is a composite measure developed using six variables of material circumstances. Deriving the material deprivation index by assigning the same weight to each individual variable may mask socioeconomic patterns existing in the COVID epidemic (18).

In this study, a high level of mobility was associated with an increased risk of COVID-19 incidence, consistent with recent studies from the United States. However, this study results are in contrast to previous studies in terms of which areas are more mobile. In most previous studies, socioeconomically disadvantaged areas were reportedly more likely to have higher mobility (9, 10); however, this study results showed that a high level of mobility was characteristic of affluent areas in Korea. This finding is understandable because mobility using public transport is concentrated in densely populated areas in the capital and large cities in Korea and within-city mobility is distributed across places of social gatherings and business meetings.

High mobility observed in affluent areas may offer another plausible explanation as to why socioeconomic inequalities differ based on the measure. Collectively, area-level socioeconomic disadvantages concerning COVID-19 incidence were mixed with lower economic activity in poor communities. Notably, when simultaneously adjusted for mobility, SES measures of basic livelihood security recipients and the nonemployment rate remained significant in the low phase but not in the rebound phase. This result indicates that mobility is a major contributing factor to the association between area-level SES and COVID-19 incidence in the rebound phase, but mobility alone does not fully explain the association; other vulnerabilities (e.g., a larger poor older population) are likely to be involved.

The area-level socioeconomic effect was stronger in the low phase, when the prevalence of COVID-19 was low, with less strict governmental measures, indicating that the area-level socioeconomic gradient is less likely to affect the variation in COVID-19 occurrence. Hypothetically, the socioeconomic inequalities in COVID-19 incidence were not exacerbated in the rebound phase. A larger inequality in the low phase may be attributed to people in poor communities being less responsive to an initial spread of COVID-19 when government public health measures were not sufficiently implemented nationwide. With progression to a widespread stage (rebound phase), the Korean government launched the testing and contact tracing system as a key part of the control strategy. The relatively effective performance of the strong government measures, with public compliance, applied in a nondiscriminatory manner, irrespective of SES, led to subsequent improvement in regional variations in incidence.

The strength of this study includes the use of nationwide incidence data and various socioeconomic measures. In particular, concurrent use of SES measures with mobility measures enabled us to obtain a better-fitted model and identify any existing associations. This study had several limitations. First, the mobility measure was obtained from the previous year and does not reflect the mobility changes induced by the COVID-19 pandemic. However, to some degree, the use of previous mobility data may serve as a proxy indicator in this interpretation because mobility change depending on SES appears minimal in Korea. The only study in which the average mobility patterns were compared during the COVID-19 period in Korea showed no significant change in mobility shaped by socioeconomic differences (38). Second, the findings in this study are limited to area-level interpretation, due to the inherent nature of ecological studies, which could not be directly applied at an individual level. Third, the variables associated with living conditions, such as poor hygiene conditions and overcrowding, could not be included due to data availability, although this would be relevant information regarding the association between SES and COVID-19 incidence. Furthermore, it is noteworthy to investigate the impact of inequalities on the incidence of COVID-19 in countries with a relatively lower number of cases and during the post-vaccination period to understand the direct effect of SES disparity on the infection adjusted for vaccination coverage.

In conclusion, COVID-19 does not occur randomly but follows socioeconomic patterns; socioeconomic inequalities in COVID-19 incidence occur concerning the unique context of a society in response to the pandemic. Despite similar contexts, each SES measure represents a specific factor and has a different ability to identify socioeconomic stratification caused by COVID-19. In Korea, where government control measures were effectively applied, with high compliance and with relatively low incidence, SES measures, such as basic livelihood security recipients, reflecting age stratification, may be preferable. Mobility was associated with COVID-19 incidence and partly explains the correlation between area-level SES and COVID-19 incidence during a high incidence period in countries such as Korea, where mobility is characteristic of affluent areas. The results confirm the necessity for emergency policy priorities concerning the older population in disadvantaged areas, including faster vaccination, and underscore a further need for socioeconomic support, including emergency relief funds.

## Data Availability Statement

The original contributions presented in the study are included in the article/Supplementary Material, further inquiries can be directed to the corresponding author.

## Author Contributions

D-sY conceived and designed the study. D-sY and MK acquired the data and wrote the original draft. MS and N-KS supported data collection. MH contributed data standardization. D-sY performed analyses. BC, MH, MS, N-KS, and SK edited the subsequent drafts. All authors read and approved the final manuscript.

## Funding

MS and MK were supported by a research grant of the Korea Health Technology R&D Project through the Korea Health Industry Development Institute (KHIDI), funded by the Ministry of Health & Welfare, Republic of Korea (grant number: HI19C1320).

## Conflict of Interest

The authors declare that the research was conducted in the absence of any commercial or financial relationships that could be construed as a potential conflict of interest.

## Publisher's Note

All claims expressed in this article are solely those of the authors and do not necessarily represent those of their affiliated organizations, or those of the publisher, the editors and the reviewers. Any product that may be evaluated in this article, or claim that may be made by its manufacturer, is not guaranteed or endorsed by the publisher.

## References

[1] Wang T Tang C Chen R Ruan H Liang W Guan W. Clinical features of coronavirus disease 2019 patients with mechanical ventilation: a nationwide study in China. Crit Care Med. (2020). 10.1097/CCM.0000000000004473 32618693 PMC7314346

[2] Hilton J Keeling MJ. Estimation of country-level basic reproductive ratios for novel Coronavirus (SARS-CoV-2/COVID-19) using synthetic contact matrices. PLoS Comput Biol. (2020) 16:e1008031. 10.1371/journal.pcbi.1008031 32614817 PMC7363110

[3] World Health Organization. WHO Coronavirus (COVID-19) (2021). Available online at: https://covid19.who.int/ (accessed December 17, 2021).

[4] Park SY Kim Y-M Yi S Lee S Na B-J Kim CB. Early release-coronavirus disease outbreak in call center, South Korea. Emerg Infect Dis. (2020) 26:1666–70. 10.3201/eid2608.201274 32324530 PMC7392450

[5] Bambra C Riordan R Ford J Matthews F. The COVID-19 pandemic and health inequalities. J Epidemiol Community Health. (2020) 74:964–8. 10.1136/jech-2020-214401 32535550 PMC7298201

[6] Abedi V Olulana O Avula V Chaudhary D Khan A Shahjouei S. Racial, economic, and health inequality and COVID-19 infection in the United States. J Racial Ethn. (2020) 1–11. 10.1101/2020.04.26.20079756 32875535 PMC7462354

[7] Chaudhry R Dranitsaris G Mubashir T Bartoszko J Riazi S. A country level analysis measuring the impact of government actions, country preparedness and socioeconomic factors on COVID-19 mortality and related health outcomes. EClinicalMedicine. (2020) 25:100464. 10.1016/j.eclinm.2020.100464 32838237 PMC7372278

[8] Kawachi I. COVID-19 and the 'rediscovery' of health inequities. Int J Epidemiol. (2020) 49:1415–8. 10.1093/ije/dyaa159 32974663 PMC7543525

[9] Chang S Pierson E Koh PW Gerardin J Redbird B Grusky D. Mobility network models of COVID-19 explain inequities and inform reopening. Nature. (2020). 10.1038/s41586-020-2923-3 33171481

[10] Weill JA Stigler M Deschenes O Springborn MR. Social distancing responses to COVID-19 emergency declarations strongly differentiated by income. Proc Natl Acad Sci U S A. (2020) 117:19658–60. 10.1073/pnas.2009412117 32727905 PMC7443940

[11] Moss JL Johnson NJ Yu M Altekruse SF Cronin KA. Comparisons of individual-and area-level socioeconomic status as proxies for individual-level measures: evidence from the Mortality Disparities in American Communities study. Popul Health Metr. (2021) 19:1–10. 10.1186/s12963-020-00244-x 33413469 PMC7792135

[12] Whittle RS Diaz-Artiles A. An ecological study of socioeconomic predictors in detection of COVID-19 cases across neighborhoods in New York City. BMC Med. (2020) 18:1–17. 10.1186/s12916-020-01731-6 32883276 PMC7471585

[13] Sen-Crowe B Lin I-C Alfaro R Mckenney M Elkbuli A. COVID-19 fatalities by zip codes and socioeconomic indicators across various US regions. Annals of Medicine and Surgery. (2021) 102471. 10.1016/j.amsu.2021.102471 34150208 PMC8196232

[14] Khanijahani A. Racial, ethnic, and socioeconomic disparities in confirmed COVID-19 cases and deaths in the United States: a county-level analysis as of November 2020. Ethn Health. (2021) 26:22–35. 10.1080/13557858.2020.1853067 33334160

[15] Sun Y Hu X Xie J. Spatial inequalities of COVID-19 mortality rate in relation to socioeconomic and environmental factors across England. Science of The Total Environment. (2021) 758:143595. 10.1016/j.scitotenv.2020.143595 33218796 PMC7664354

[16] Hatef E Chang H-Y Kitchen C Weiner JP Kharrazi H. Assessing the impact of neighborhood socioeconomic characteristics on COVID-19 prevalence across seven states in the United States. Frontiers in public health. (2020) 8. 10.3389/fpubh.2020.571808 33072710 PMC7536340

[17] Khan KS Torpiano G Mclellan M Mahmud S. The impact of socioeconomic status on 30-day mortality in hospitalized patients with COVID-19 infection. J Med Virol. (2021) 93:995–1001. 10.1002/jmv.26371 32729937

[18] Pickett KE Pearl M. Multilevel analyses of neighbourhood socioeconomic context and health outcomes: a critical review. Journal of Epidemiology & Community Health. (2001) 55:111–22. 10.1136/jech.55.2.111 11154250 PMC1731829

[19] Garnier R Benetka JR Kraemer J Bansal S. Socioeconomic disparities in social distancing during the COVID-19 pandemic in the United States: observational study. J Med Internet Res. (2021) 23:e24591. 10.2196/24591 33351774 PMC7837167

[20] Lee WD Qian M Schwanen T. The association between socioeconomic status and mobility reductions in the early stage of England's COVID-19 epidemic. Health Place. (2021) 69:102563–102563. 10.1016/j.healthplace.2021.102563 33799134 PMC9673007

[21] Balmford B Annan JD Hargreaves JC Altoe M Bateman IJ. Cross-country comparisons of covid-19: policy, politics and the price of life. Environ Resour Econ (Dordr). (2020) 1–27. 10.1007/s10640-020-00466-5 32836862 PMC7400753

[22] Kang S-J Kim S Park K-H Jung SI Shin M-H Kweon S-S. Successful control of COVID-19 outbreak through tracing, testing, and isolation: Lessons learned from the outbreak control efforts made in a metropolitan city of South Korea. J Infect Public Health. (2021) 14:1151–4. 10.1016/j.jiph.2021.07.003 34364306 PMC8276554

[23] Kim S Castro MC. Spatiotemporal pattern of COVID-19 and government response in South Korea (as of May 31, 2020). Int J Infect Dis. (2020) 98:328–33. 10.1016/j.ijid.2020.07.004 32634584 PMC7334954

[24] Yun J-W Kim Y-J Son M. Regional deprivation index and socioeconomic inequalities related to infant deaths in Korea. J Korean Med Sci. (2016) 31:568–78. 10.3346/jkms.2016.31.4.568 27051241 PMC4810340

[25] Jones SR Riddell WC. Unemployment and Non-employment Heterogeneities in Labour Market States. Department of Economics, University of British Columbia. (2002).

[26] Moreno-Betancur M Latouche A Menvielle G Kunst AE Rey G. Relative index of inequality and slope index of inequality: a structured regression framework for estimation. Epidemiology. (2015) 26:518–27. 10.1097/EDE.0000000000000311 26000548

[27] Getis A Aldstadt J. Constructing the spatial weights matrix using a local statistic. Geogr Anal. (2004) 36:90–104. 10.1111/j.1538-4632.2004.tb01127.x

[28] Gelman A Carlin JB Stern HS Dunson DB Vehtari A Rubin DB. Bayesian Data Analysis, 3rd ed. Chapman and Hall/CRC (2013). 10.1201/b16018

[29] Lunn DJ Thomas A Best N Spiegelhalter D. WinBUGS-a Bayesian modelling framework: concepts, structure, and extensibility. Stat Comput. (2000) 10:325–37. 10.1023/A:1008929526011

[30] National Geographic Information Institute South Korea. South Korea Map (2016). Available online at: http://map.ngii.go.kr/ms/map/NlipMap.do?tabGb=statsMap (accessed July 10, 2021).

[31] Sergeant JC Firth D. (2006). Relative index of inequality: definition, estimation, and inference. Biostatistics. 7:213–4. 10.1093/biostatistics/kxj002 16192414

[32] Luo Y Yan J Mcclure S. Distribution of the environmental and socioeconomic risk factors on COVID-19 death rate across continental USA: a spatial nonlinear analysis. Environmental Science and Pollution Research. (2021) 28:6587–99. 10.1007/s11356-020-10962-2 33001396 PMC7527667

[33] Aguilar-Palacio I Maldonado L Malo S Sánchez-Recio R Marcos-Campos I Magallón-Botaya R. COVID-19 Inequalities: Individual and Area Socioeconomic Factors (Aragón, Spain). Int J Environ Res Public Health. (2021) 18:6607. 10.3390/ijerph18126607 34205348 PMC8296401

[34] Politi J Martín-Sánchez M Mercuriali L Borras-Bermejo B Lopez-Contreras J Vilella A. Epidemiological characteristics and outcomes of COVID-19 cases: mortality inequalities by socio-economic status, Barcelona, Spain, 24 February to 4 May 2020. Eurosurveillance. (2021) 26:2001138. 10.2807/1560-7917.ES.2021.26.20.2001138 34018483 PMC8138960

[35] Jeong HE Lee J Shin HJ Shin J-Y. Socioeconomic disparities in Korea by health insurance type during the COVID-19 pandemic: a nationwide study. Epidemiol Health. (2021) 43. 10.4178/epih.e2021007 33445821 PMC8060526

[36] Briggs AD Fraser C. Is NHS Test and Trace exacerbating COVID-19 inequalities? Lancet. (2020) 396:1972. 10.1016/S0140-6736(20)32593-9 33285140 PMC7834366

[37] Oh TK Choi J-W Song I-A. Socioeconomic disparity and the risk of contracting COVID-19 in South Korea: an NHIS-COVID-19 database cohort study. BMC Public Health. (2021) 21:1–12. 10.1186/s12889-021-10207-y 33451306 PMC7809637

[38] Lee M Lee S Kim S Park N. (2020). Human Mobility during COVID-19 in the Context of Mild Social Distancing: Implications for Technological Interventions. arXiv preprint arXiv:2006.16965.

