# Supplementary material for: Socioeconomic Inequalities in COVID-19 Incidence During Different Epidemic Phases in South Korea
Source: Front Med (Lausanne). 2022 Mar 8;9:840685. doi: 10.3389/fmed.2022.840685 (PMC8957264; doi:10.3389/fmed.2022.840685)
Supplement: Supplementary file 1 [file Data_Sheet_1.docx]

Supplementary Material

**Methods and Materials**

1. Relative index of inequality

Relative index of inequality (RII) was estimated to measure inequality in COVID-19 incidence rate at the area-level. To determine socio-economic strata of SES measures, the continuous values of socio-economic factors were converted into quintiles of their distribution (i.e. each stratum accounted for 20% of the number of municipalities). RII is a commonly used measure of health inequality that summarises the distribution of a health outcome measure against a socio-economic status as the relative difference in a health outcome during a certain period of time within a larger group of the least and most deprived subgroups. RII in this study corresponded to the relative risk of COVID-19 incidence in the most socio-economically deprived municipalities compared with the least deprived municipalities.

2. Bayesian Regression

The models were iterated up to the point where convergence was considered to have been achieved based on a visual inspection of MCMC trace plots and Gelman-Rubin convergence diagnostic. The models were run with three chains with different starting values. The first 4,000 iterations were discarded, and the remaining parameter values comprised the posterior distributions. After the model run, we estimated the posterior distribution of parameters for socio-economic status and covariates. BYM model was used to to account for spatial autocorrelation of residuals, calculated by averaging neighbouring random effects and extra residual term to account for spatially independent variation that is independent, identical and normal distributed as followed.

$$Y_{i}\sim NB\left( \pi_{i},r_{i} \right),Y_{i}: Number of COVID-19 cases by municipality i$$

$\pi_{i}=\frac{r_{i}}{r_{i}+\lambda_{i}},E\left( Y_{i} \right)=\lambda_{i}$

$$log\left( \lambda_{i} \right)={\alpha+log\left( \mathrm{populatio}n_{i} \right)+\beta}_{1}\times{SES}_{i,k}+\sum_{j=2}^{N} \beta_{j}\times covariate_{i}+u_{i}+\varepsilon_{i}$$

$$u_{1:229}\sim ICAR\left( W,\sigma_{u}^{2} \right)$$

$$\varepsilon\sim N\left( 0,\sigma_{\varepsilon}^{2} \right)$$

$u \sim N(0, (I-{C)}^{-1} \times M$), $C=\gamma\times W,M=I\times$ $\sigma_{u}^{2}$

The u is spatial correlated random effect, calculated by averaging neighbouring random effects, where I is identity matrix, and W is spatial weights matrix, constructed by a inverse distance function with the expoents 2, $\frac{1}{\left( d_{ij} \right)^{2}}$, dij is equal to distance between municipality i and j,followed by row- standardized such that each of row sums to 1 for interpretation of the parameters (Getis & Aldstadt, 2004). Spatial correlation parameter, which is denoted as $\gamma$set to 1.

Uniform prior was used as a prior distribution for alpha, overall fixed effect (Besag & Kooperberg, 1995) , and for parameters for socio-economic status and covariates. Gamma distribution with shape and inverse scale parameters both equal to 0.01, which correspond to a mean of 1 and a variance of 100, used as a prior distribution for a variance for non-spatially structured residual term. Gamma prior(0.5, 0.0005) prior for the precision parameter of the spatial random effect was used to embrace for the situations where spatial dependency between municipalities is negligible(Kelsall & Wakefield, 1999).

$$\alpha\sim Uniform\left( -\infty,\infty\right)$$

$$\beta\sim Uniform\left( -10,10 \right)$$

$$\sigma_{u}^{2}\sim gamma\left( 0.5,0.0005 \right)$$

$$\sigma_{\varepsilon}^{2}\sim gamma\left( 0.01,0.01 \right)$$

**Results**

| **Table S1** Incidence inequality of COVID-19 by each socioeconomic status and activity over the low and rebound phase at municipal level | | | | | | |
| --- | --- | --- | --- | --- | --- | --- |
| Variables | Epidemic Phase | | | | | |
|  | Low phase  (no. of cases =2,906) | | | Rebound phase  (no. of cases =40,545) | | |
|  | Model 1 † | Model 2 ‡ | Model 3§ | Model 1 † | Model 2 ‡ | Model 3§ |
| Socioeconomic status |  |  |  |  |  |  |
| National insurance contributions | 0.48  (0.21,1.03) | 0.61  (0.20,1.90) | - | 0.81  (0.54,1.21) | 1.00  (0.99,1.01) | - |
| Material deprivation index | 0.84 (0.42, 1.59) | 0.56 (0.18, 1.86) | - | 0.90 (0.63, 1.26) | 1.01 (0.61, 1.67) | - |
| Non-employment rate | 2.30 (1.33, 4.03) | 1.40 (1.01, 1.95) | 2.67 (1.35, 5.50) | 0.99 (0.73, 1.35) | 4.20 (1.97, 9.12) | 1.02 (0.75, 1.40) |
| Basic livelihood security recipient | 1.50 (1.06, 2.02) | 2.66  (1.12, 5.97) | 2.22 (1.05, 4.62) | 1.11  (1.02, 1.22) | 1.06  (1.01, 1.12) | 1.21 (0.88, 1.65) |
| Financial autonomy | 1.24  (0.60, 2.55) | 2.92  (0.97, 8.35) | - | 0.81  (0.57, 1.16) | 1.00 (0.99, 1.00*) | - |
| Economic activity |  |  |  |  |  |  |
| Mobility at risk | 10.77  (3.76, 31.88) | 1.66  (1.26, 2.17) | - | 1.41  (0.82, 2.44) | 1.28  (1.14, 1.44) | - |
| The relative index of inequality (RII) was obtained using a spatial negative binomial model with marten correlation function for spatial correlation term, 95% confidence interval was estimated by bootstrap, denoted in parenthesis. RII corresponds to the relative risk of the incidence for COVID-19 in the lowest socioeconomic regions over in the highest socioeconomic regions.  † Model 1: unadjusted model  ‡ Model 2: adjusted for three covariates (human density, median age, and health care workforce)  § Model 3: Model 2 + socioeconomic activity (mobility) at municipality levels; Model 3 remained only significant variables at the low phase in model 2. | | | | | | |

**References**

Besag, J., & Kooperberg, C. (1995). On conditional and intrinsic autoregressions. *Biometrika, 82*(4), 733-746.

Getis, A., & Aldstadt, J. (2004). Constructing the spatial weights matrix using a local statistic. *Geographical analysis, 36*(2), 90-104.

Kelsall, J., & Wakefield, J. (1999). Discussion of ‘Bayesian models for spatially correlated disease and exposure data’, by Best et al. *Bayesian statistics, 6*, 151.
